# Nano-Hydroxyapatite Derived from Biogenic and Bioinspired Calcium Carbonates: Synthesis and In Vitro Bioactivity

**DOI:** 10.3390/nano11020264

**Published:** 2021-01-20

**Authors:** Francesca Cestari, Francesca Agostinacchio, Anna Galotta, Giovanni Chemello, Antonella Motta, Vincenzo M. Sglavo

**Affiliations:** 1 Department of Industrial Engineering, University of Trento, Via Sommarive 9, 38123 Trento, Italy; f.agostinacchio@unitn.it (F.A.); anna.galotta@unitn.it (A.G.); giovannichemello@gmail.com (G.C.); antonella.motta@unitn.it (A.M.); vincenzo.sglavo@unitn.it (V.M.S.); 2 BIOTech Research Center, and European Institute of Excellence on Tissue Engineering and Regenerative Medicine Unit, University of Trento, via delle Regole 101, 38123 Trento, Italy; 3 INSTM, Via G. Giusti 9, 50121 Firenze, Italy

**Keywords:** calcium orthophosphates, nano-hydroxyapatite, eggshell, cuttlefish bone, mussel shell, amorphous calcium carbonate

## Abstract

Biogenic calcium carbonates naturally contain ions that can be beneficial for bone regeneration and therefore are attractive resources for the production of bioactive calcium phosphates. In the present work, cuttlefish bones, mussel shells, chicken eggshells and bioinspired amorphous calcium carbonate were used to synthesize hydroxyapatite nano-powders which were consolidated into cylindrical pellets by uniaxial pressing and sintering 800–1100 °C. Mineralogical, structural and chemical composition were studied by SEM, XRD, inductively coupled plasma/optical emission spectroscopy (ICP/OES). The results show that the phase composition of the sintered materials depends on the Ca/P molar ratio and on the specific CaCO_3_ source, very likely associated with the presence of some doping elements like Mg^2+^ in eggshell and Sr^2+^ in cuttlebone. Different CaCO_3_ sources also resulted in variable densification and sintering temperature. Preliminary in vitro tests were carried out (by the LDH assay) and they did not reveal any cytotoxic effects, while good cell adhesion and proliferation was observed at day 1, 3 and 5 after seeding through confocal microscopy. Among the different tested materials, those derived from eggshells and sintered at 900 °C promoted the best cell adhesion pattern, while those from cuttlebone and amorphous calcium carbonate showed round-shaped cells and poorer cell-to-cell interconnection.

## 1. Introduction

A material is defined as bioactive, or biologically active, when it is capable of generating an interphase bonding layer across the material–tissue interface, thus improving the ability to bond directly with the living structure. One strategy to attain bioactivity in ceramic materials is to mimic the chemistry of the bone tissue and this is why calcium phosphates and, in particular, hydroxyapatite (HA, Ca_10_(PO_4_)_6_OH_2_), have been widely studied for bone regeneration applications. Nevertheless, the main inorganic constituent of human bone is substantially different from pure HA, as it is a nanocrystalline non-stoichiometric compound containing sodium, magnesium and carbonate ions together with significant amounts of other trace elements such as K^+^, F^−^, Cl^−^, Zn^2+^, Sr^2+^, Ba^2+^, etc. For this reason, it should be more properly referred to as “impure hydroxyapatite” [1] or “biological apatite” [2]. The presence of the said additional elements has been recognized to play an important role in bone repair [3] and, for example, Mg^2+^ and Sr^2+^ have been found to improve osteoblasts adhesion and bone formation [4,5,6,7,8]. The impurities, especially of CO_3_^2−^, also introduce a certain degree of disorder into the crystal lattice that increases the solubility of the material and, therefore, its resorbability [9].

The incorporation of additional chemical elements in synthetic HA may be an expensive process, but it can be more compliant if hydroxyapatite is extracted from biological resources like mammalian bones, fish bones or biogenic calcium carbonates, which are usually in the form of calcite or aragonite [10]. Cuttlebone-derived aragonite was used to produce HA by several authors [11,12,13,14,15,16,17] after the pioneering work by Rocha et al. [18,19] who observed good in vitro bioactivity of HA scaffolds derived from cuttlefish bones. Other biological sources of calcium carbonate used to synthesize HA are eggshells [20,21,22,23,24,25,26,27,28], corals [29,30,31], algae [32] and sea shells [33,34,35,36,37,38,39]. Most of these studies report the synthesis of nanocrystalline calcium-deficient hydroxyapatite (CDHA), with significant levels of carbonate ions and other impurities, like Mg in eggshell-derived HA [40]. Another form of calcium carbonate that is found in biological systems is amorphous calcium carbonate (ACC), which can be stable or can be a transient precursor for the formation of calcite or aragonite [41], as in the case of sea urchin spines [42] and mollusk larval shells [43]. Due to its role in bio-mineralization, ACC is a promising material for bone tissue engineering, but its applicability in this field was considered in only a few studies [44,45] and, to the authors’ knowledge, not for the synthesis of HA.

Although the bioactivity of naturally derived calcium phosphates has been proven by in vitro [46,47] and in vivo [48,49] biological tests, the role of calcium carbonate precursors on the biological behavior of HA is still not completely clear. Kim et al. [50] found that HA granules derived from cuttlebone promote superior cell proliferation and differentiation in vitro with respect to synthetic HA and stimulate more new bone formation in calvarial defects in white rabbits. Nevertheless, they filled the defects directly with HA granules while HA powder usually needs to be consolidated by a sintering process to develop minimal mechanical properties.

The sintering of pure HA is usually carried out at temperatures between 1100 °C and 1250 °C [51], whereas carbonate ion substitutions can lower the densification temperature to 900–950 °C [52]. Unfortunately, this thermal treatment may lead to the loss of some important properties that are thought to be beneficial for bioactivity: the carbonate content can be reduced, the nano-crystallinity lost and CDHA transformed into stoichiometric HA and β-TCP [53]. In this respect, the bioactivity of sintered synthetic HA and fish bone-derived HA was compared by Mondal et al. [54], who found that cell viability is basically the same, although osteoblasts seem to be better attached to the fish-HA than to the synthetic one.

In the present work, we synthesized HA nanopowders, starting from four different calcium carbonate sources: chicken eggshells (biogenic calcite), cuttlefish bones (biogenic aragonite), mussel shells (biogenic calcite/aragonite) and synthetic amorphous calcium carbonate (bioinspired ACC). The powders were then consolidated by uniaxial pressing and sintering at 800–1100 °C and the materials, carefully characterized, were subjected to cytotoxicity and in vitro cell adhesion tests to point out and compare their bioactivity for potential use in bone tissue engineering.

## 2. Materials and Methods

Chicken eggshells (ES), cuttlefish (*sepia officinalis*) bones (CB) and mussel (*mytilus galloprovincialis*) shells (MS) were collected as food waste from a local bakery, fish shop and restaurant, respectively. After washing under tap water, they were boiled in demineralized water for 10 min, dried overnight at 100 °C, ground to powder in a centrifugal mill (Retsch S100) at 400 rpm for 30 min and then sieved in order to eliminate particles larger than 300 µm. The amorphous calcium carbonate (ACC) was kindly supplied by Amorphical (Harash St. 11, Nes-Ziona, Israel) as a synthetic powder with the commercial name DENSITY™. Pure stoichiometric HA granules (sHA), powder size 5–25 µm, were achieved by S.A.I. (Science Application Industry, Saint-Priest, France).

Hydroxyapatite was synthesized from ES, CB, MS and ACC powders via wet mechanosynthesis and successive drying in an oven. The powders were mixed with ammonium phosphate dibasic ((NH_4_)_2_HPO_4_, CAS: 7783-28-0, purchased from Fluka, Buchs, Switzerland) or phosphoric acid (~85% H_3_PO_4_, CAS: 7664-38-2, purchased from CARLO ERBA Reagents, Cornaredo, Italy), in order to achieve a Ca/P ratio of 1.67. The mechanosynthesis reaction was promoted by ball-milling the reactants in an aqueous solution, using a Turbula^®^ mixer and a 250 mL polyethylene bottle filled with zirconia balls (ball mass = 0.5 g), with balls-to-powder mass ratio equal to 10:1. The resulting slurry was dried in an oven for 24 h.

In a previous work the effect of the pH, the milling time and the drying temperature on the mechanosynthesis process efficiency was investigated [55]. According to the obtained results, the process parameters listed in Table 1 were selected in order to maximize the efficiency and minimize the processing time and temperature. Therefore, CB and ACC, that were more prone to be converted into HA, were ball-milled for 30 min and dried at 120 °C, while ES and MS needed 4 h milling and 150 °C drying temperature. We used (NH_4_)_2_HPO_4_ as the phosphate provider for CB, MS and ACC, while ES were processed with H_3_PO_4_ to facilitate the reaction by the dissolution of CaCO_3_.

The as-synthesized HA powders were then consolidated by uniaxial pressing and sintering. About 0.1 g of each powder was pressed with 2 tons in a 5 mm diameter cylindrical die using a Specac manual hydraulic press. The pellets were heated at 10 °C/min in a muffle furnace (Nabertherm P330), maintained at the selected sintering temperature for 2 h and free cooled in the oven. The sintering temperature was set for each material after some preliminary dilatometric tests on the green pellets, in order to densify the powders while retaining some porosity. The sintering temperature was set to 800 °C for ACC–HA, 900 °C for ES–HA, 1000 °C for MS–HA and 1100 °C for sHA. For CB–HA two temperatures were selected, 900 °C and 1100 °C. For practical convenience, the final materials were named after the calcium carbonate precursor and the sintering temperature as follows: ACC-800, ES-900, MS-1000, sHA-1100, CB-900 and CB-1100.

The crystalline phases of calcium precursors, as-synthesized powders and sintered pellets were characterized by x-ray diffraction (XRD). The CaCO_3_ powders spectra were acquired in reflection geometry with an Italstructures IPD3000 X-ray diffractometer, equipped with a Co anode source (Kα radiation 1.78892 Å), a multilayer monochromator to suppress k-beta radiation, fixed 100 µm slits and an Inel CPS120 detector over 5–120° 2-theta range (0.03 degrees per channel). The as-synthesized powders and sintered pellets, instead, were analyzed using a Rigaku IIID-max, Cu anode source (Kα radiation 1.5406 Å), 5–110° 2-theta range, scan step 0.05° and step time 2 s. The spectra were then analyzed with the Rietveld software Maud [56], using the following crystal phases downloaded from the database COD [57]: calcite n. 4,502,443 [58], aragonite n. 2,100,187 [59], HA n. 4,317,043 [60], β-TCP n. 1,517,238 [61], CPP (Ca_2_P_2_O_7_) n. 1,001,556 [62], CaO n. 9,006,712 [63] and CaOH n. 1,000,045 [64].

The Ca and P content and the presence of other elements in the calcium carbonate precursors and in the as-synthesized powders were determined with inductively coupled plasma/optical emission spectroscopy (ICP/OES), using Spectro Ciros Vision CCD (125–770 nm) and hydroxyapatite ultrapure standard (>99.995% trace metal basis, Sigma–Aldrich, St. Louis, MO, USA). The samples were solubilized in ultrapure nitric acid (70 vol%, Sigma–Aldrich, St. Louis, MO, USA) and diluted with pure water (obtained by reverse osmosis, σ < 0.1 μS cm^−1^). The emission lines chosen for the analysis were 184 nm for Ca and 178 nm for P. The analyses of the other elements shall be considered as semi-quantitative, only.

The internal porosity and surface morphology of the sintered pellets were observed with a JEOL JSM-5500 scanning electron microscope (SEM) using secondary electrons, after Pt/Pd metallization with a QuorumQ150TES sputtering equipment. The density of the sintered pellets was estimated by weighting and measuring 5 samples per type, using a caliper and a laboratory scale.

The cytotoxicity of the sintered pellets was evaluated using human lung fibroblasts cell line (MRC5), expanded and cultured under standard conditions (37 °C and 5% CO_2_) in minimal essential medium (MEM—Gibco, Thermo Fisher Scientific, Waltham, MA, USA), 10% of inactivated fetal bovine serum (Euroclone, Pero, Italy), supplemented with 1% of L-glutamine (Euroclone, Pero, Italy), sodium pyruvate (Gibco, Thermo Fisher Scientific, Waltham, MA, USA), non-essential amino acids (SigmaAldrich, St. Louis, MO, USA), and antibiotic-antimycotic (Euroclone, Pero, Italy). All samples were sterilized by autoclave. LDH cytotoxic assay (Thermo Fisher Scientific, Waltham, MA, USA) was performed to measure the amount of lactate dehydrogenase (LDH) released by cells during cell death. The test was performed following the European Standard EN ISO-10993-12:2004 and 10993-5:2009. Specifically, all samples without cells were incubated in medium without phenol red and with heat inactivated serum for 72 h (conditioned medium). After incubation, the conditioned medium was pursed on MRC5 cells at 70% of confluence, previously seeded at a density of 5000 cell/well in a 96-well plate and incubated for 48 h. Positive and negative controls were represented by fully lysate cells and cells cultured in standard medium, respectively. After the incubation, all samples were prepared following the manufacturer’s instructions and the LDH amount released in the medium was measured using a Tecan Infinite 200 microplate reader (Tecan Group, Männedorf, Switzerland), recording the absorbance at 490 nm and the background at 680 nm. Five replicates were tested per each condition.

Cell adhesion was evaluated by confocal analysis (A1 Laser Microscope, Nikon Instruments Europe BV, Amsterdam, The Netherlands) using a human osteosarcoma cell line (MG63). The sterilized sintered pellets were placed in a 96-well culture plate and seeded with 6000 cells/well. The cells were cultured at 37 °C with 5% CO_2_ in a culture media composed of MEM (Gibco, Thermo Fisher Scientific, Waltham, MA, USA), 10% fetal bovine serum (Euroclone, Pero, Italy), 1% sodium pyruvate (Gibco, Thermo Fisher Scientific, Waltham, MA, USA), 1% non-essential amino acids (Sigma-Aldrich, St. Louis, MO, USA), 1% L-glutamine (Euroclone, Pero, Italy) and 1% of antibiotic/antimycotic (Euroclone, Pero, Italy). All samples were observed at day 1, 3, and 5 after seeding. Before the confocal analysis, the specimens were fixed with 4% paraformaldehyde for 40 min at each time point, and later cell membranes were permeabilized with Triton X-100 at 0.2%. The cellular nuclei and the cytoskeleton were stained with 4, 6 diamidino 2 phenylindole, dilactate (DAPI—SigmaAldrich, St. Louis, MO, USA) and I-Fluor 488 (Abcam, Cambridge, UK), respectively.

## 3. Results and Discussion

### 3.1. Materials Characterization

**Figure 1:**
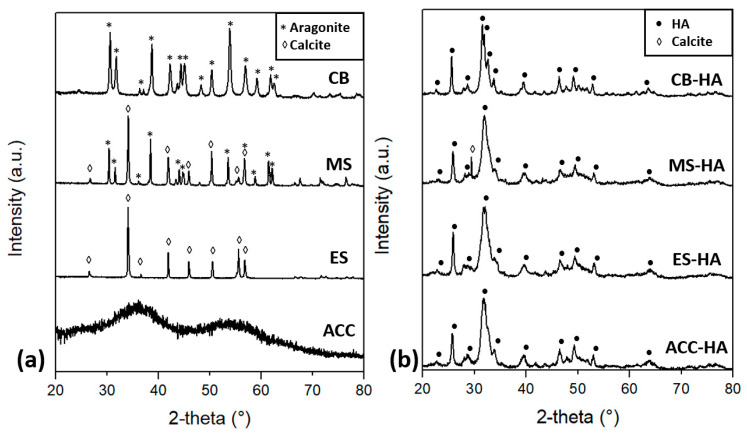
XRD spectra of the (**a**) raw materials and (**b**) synthesized powders.

The XRD spectra of the raw materials (Figure 1a) and the corresponding quantitative phase analysis (Table 2) show that eggshells (ES) are composed of 100% calcite, cuttlebones (CB) of 100% aragonite and mussel shells (MS) of a mixture of ~70/30 calcite/aragonite. The amorphous structure of ACC, instead, is revealed by the two broad bands at about 35° and 53° (Co Kα radiation) [65]. The diffraction spectra of the synthesized powders shown in Figure 1b confirm that all materials were converted into hydroxyapatite, with about 7 wt% residual calcite only in the case of MS. The width of the HA peaks indicates that the crystals are nanosized, as confirmed also by the Rietveld analyses, which pointed out crystallite sizes of 13 nm (ES–HA and MS–HA), 20 nm (ACC–HA) and 25 nm (CB–HA). In addition, the high relative intensity of the (002) peak at 25° with respect to the (211) one at 31° suggests a preferential growth along the c-axis for all materials [66].

**Figure 2:**
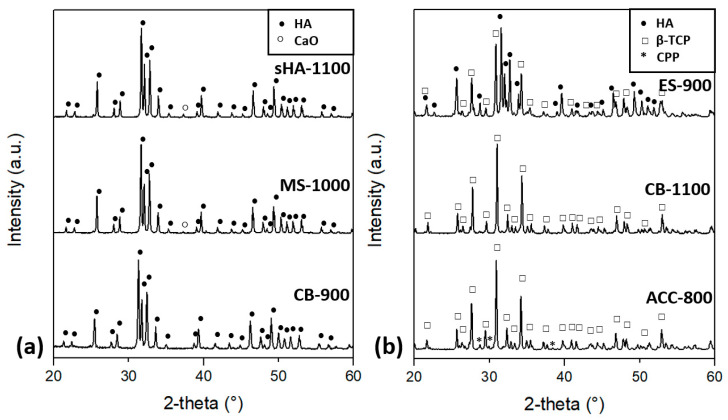
XRD spectra of the sintered pellets: (**a**) sHA-1100, MS-1000 and CB-900; (**b**) ES-900, CB-1100 and ACC-800.

The sintered pellets show a different crystal structure with respect to the synthesized powders, as shown in Figure 2 and summarized in Table 2. First of all, the crystallites dimension is no longer nanometric but they become micrometric (>200 nm). MS-1000 and CB-900 maintain the hexagonal HA structure but the preferred orientation is attenuated (MS-1000) or even completely nullified (CB-900).

The results of the ICP/OES analyses are summarized in Table 3. Significant levels of Na (1.5%) and P (~2%) were found in the raw ACC powder; Mg is present (~0.4%) in the eggshell and Sr (~0.2%) in the cuttlebone. Traces of Na, Mg and Sr were detected in all biogenic CaCO_3_, while K was found only in CB and ES. The amount of Ca and P measured in the synthesized HA powders yields to Ca/P ratio lower than 1.67 for ACC–HA, CB–HA and ES–HA, pointing out that these nanopowders are constituted by calcium deficient HA (CDHA). MS–HA and sHA, instead, are characterized by a Ca/P ratio larger than 1.67. It has to be said that, since it was not possible to completely dissolve the biogenic HA powders in nitric acid solution during the preparation of the samples, the results may slightly deviate from the reality, and for this reason the data are presented with an estimated error of ±0.02. Other very limited impurities (<50 ppm) were also found in the materials like Cl in ACC and CB; Ba in CB and ES; Zn in CB; Fe, Si, and Mn in MS.

The results of the XRD analyses can be correlated with the amount of P and Ca measured by ICP/OES in the HA powders, considering the CaO/P_2_O_5_ phase diagram [67]. In the presence of water, 100% HA is expected with Ca/P = 1.67, while 100% β-TCP is formed with Ca/P = 1.5. For a Ca/P ratio between these two values both phases are expected following the lever rule. Accordingly, ES–HA transforms from CDHA to biphasic HA/β-TCP when sintered at 900 °C (ES-900), in the proportion expected for Ca/P = 1.58, which is 50/50. Conversely, CB–HA, with Ca/P = 1.64, maintains the hexagonal HA structure after heat treatment at 900 °C (CB-900), but transforms into ~90% β-TCP, ~5% HA and ~5% CaOH when treated at 1100 °C (CB-1100).

The different behavior of eggshell- and cuttlebone-derived HA might be related to the different content of bivalent cations, with Sr^2+^ being mainly present in CB and Mg^2+^ in ES. In fact, it is known that both magnesium and strontium stabilize the crystal structure of β-TCP, at the expense of HA [68,69,70]. Nevertheless, their effect is different, because cations with ionic radii larger than Ca^2+^, such as Sr^2+^, can be incorporated in the apatite structure to a much greater extent than those with a smaller ionic radius like Mg^2+^ [1]. Magnesium is thought to inhibit apatite crystal growth, to stabilize β-TCP with respect to α-TCP [71] and to lower the temperature at which CDHA transforms into a biphasic mixture of β-TCP and HA [72]. Therefore, the presence of Mg in ES–HA could be the reason for ES–HA to transform partially into β-TCP at 900 °C while CB–HA does not. However, it is surprising that CB–HA, when sintered at 1100 °C, is composed mainly by β-TCP, when the phase diagram predicts the prevalence of HA (~85% HA). The extra amount of calcium results instead in the presence of a small amount of CaOH. It is possible that the Sr^2+^ content caused a deviation from the expected behavior by favoring the formation of β-TCP, although the reason for the CaOH formation is still unclear. One possible explanation could be the non-homogenous distribution of Ca^2+^ and PO_4_^3−^ ions, which can determine a Ca/P ratio larger than 1.67 in certain areas, leading to the formation of calcium hydroxide.

When calcium and phosphorus are present in proportions larger than 1.67, the phase diagram predicts the presence of CaO together with HA, as a result of the extra amount of calcium. Accordingly, the XRD spectra of sHA-1100 and MS-1100, whose powders are characterized by Ca/P equal to 1.71 and 1.76, respectively, revealed the presence of a small amount of CaO. Moreover, the as-synthesized MS–HA powder contains ~7% of unreacted calcite, probably because of the insufficient amount of phosphorus. 

As for ACC–HA, the measured Ca/P ratio is 1.28, which falls into a region of the phase diagram where, instead of HA, a mixture of β-TCP (Ca/P = 1.5) and CPP (calcium pyrophosphate, Ca_2_P_2_O_7_, Ca/P = 1.0) is expected [67]. Accordingly, ACC–HA converts into ~85 wt% β-TCP and ~15 wt% CPP upon sintering at 800 °C (ACC-800). Nevertheless, if we predict the Ca/P ratio based on the phase composition determined by the Rietveld analyses as in [73], we obtain Ca/P = 1.41 instead of 1.28. This discrepancy could be explained by considering that phosphorus is also present in the initial ACC raw powder (Table 3), most probably because it was added to stabilize the amorphous phase [74]. It is possible that said phosphorus content was detected by ICP/OES but it was not fully available for the formation of calcium phosphates, being, for example, in a form that is different from PO_4_^3−^ ions. This could explain the discrepancy between the Rietveld analysis and the Ca/P ratio measured by ICP/OES, taking also into account that Ca and P in CDHA are rarely in proportions lower than 1.30 [75].

**Figure 3:**
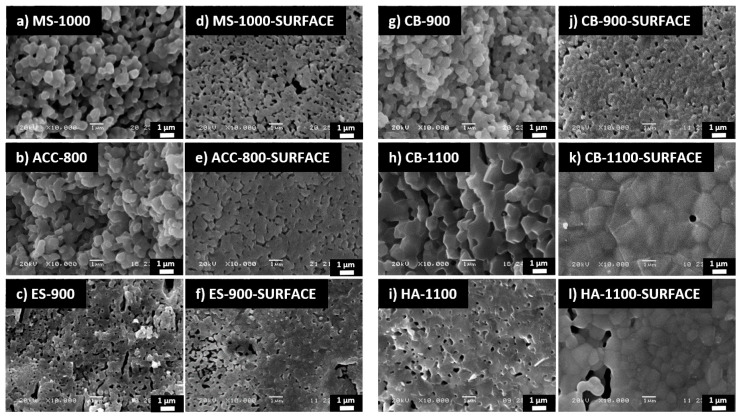
SEM images of the sintered materials: fracture surfaces (**a**–**c**,**g**–**i**); external surfaces that come into contact with the cells (**d**–**f**,**j**–**l**).

The morphology of the fracture and external surfaces of the sintered pellets are shown in Figure 3. Well-developed necks among particles are visible in all samples, although the densification is clearly not complete, as confirmed also by the density measurements reported in Table 4. As expected, CB-1100 reaches a higher densification with respect to CB-900, the relative density being ~85% and ~75%, respectively. The grains of CB-1100, visible on the material surface, look slightly bigger than those of HA-1100, even if the density is very similar. This could be correlated with the recrystallization process from CDHA to β-TCP that occurred simultaneously with the densification in CB-1100, while HA-1100 showed grain growth without recrystallization.

The density measurements and SEM analyses also show that the densification of ACC-800 is comparable to that of MS-1000 (Table 4) and its grain size is very similar to CB-900 and MS-1000 (Figure 3). The fact that ACC–HA densifies at lower temperatures with respect to biogenic HA powders could be explained by the presence of some amorphous CaCO_3_ that could favor sintering, remaining unreacted after the synthesis process, but not being clearly revealed by XRD.

The SEM micrographs also show that, for all materials, most of the pores have dimensions of about 1 µm or less, therefore it can be assumed that they are not perceived as holes by the cells, which have dimensions close to 100 µm. However, the presence of micrometric pores is thought to be beneficial for the interaction of the bioceramics with the cells [76].

### 3.2. In Vitro Biological Evaluation

**Figure 4:**
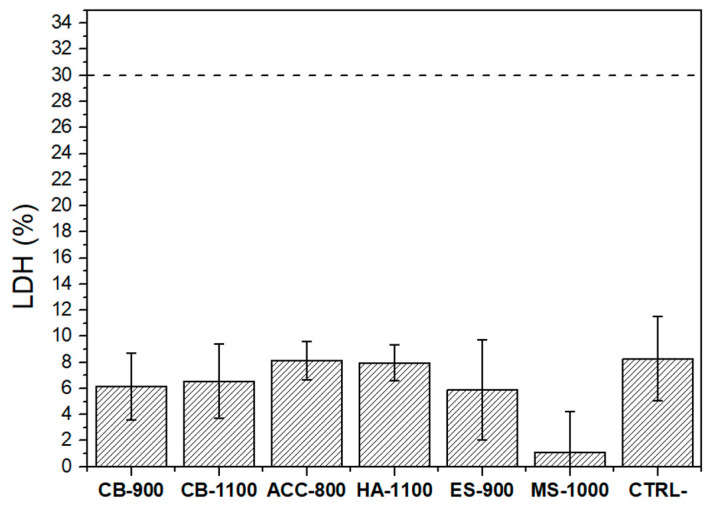
The LDH levels of the sintered pellets. The cytotoxicity threshold (30%) is indicated by the dashed line.

LDH assay was performed to evaluate potential cytotoxic effects. After 48 h incubation, the conditioned medium in contact with cells was tested and the results are shown in Figure 4. According to the European Standard EN ISO-10993-12:2004 and 10993-5:2009, samples are considered cytotoxic when the LDH amount released into the medium is equal to or above 30%. As shown, all tested conditions were below the threshold of cytotoxicity. Indeed, LDH released did not exceed 8%, demonstrating that HA derived from chicken eggshells (biogenic calcite), cuttlefish bones (biogenic aragonite), mussel shells (biogenic calcite/aragonite), and synthetic amorphous calcium carbonate (bioinspired ACC) do not exhibit any cytotoxic effects on MRC5 cells.

**Figure 5:**
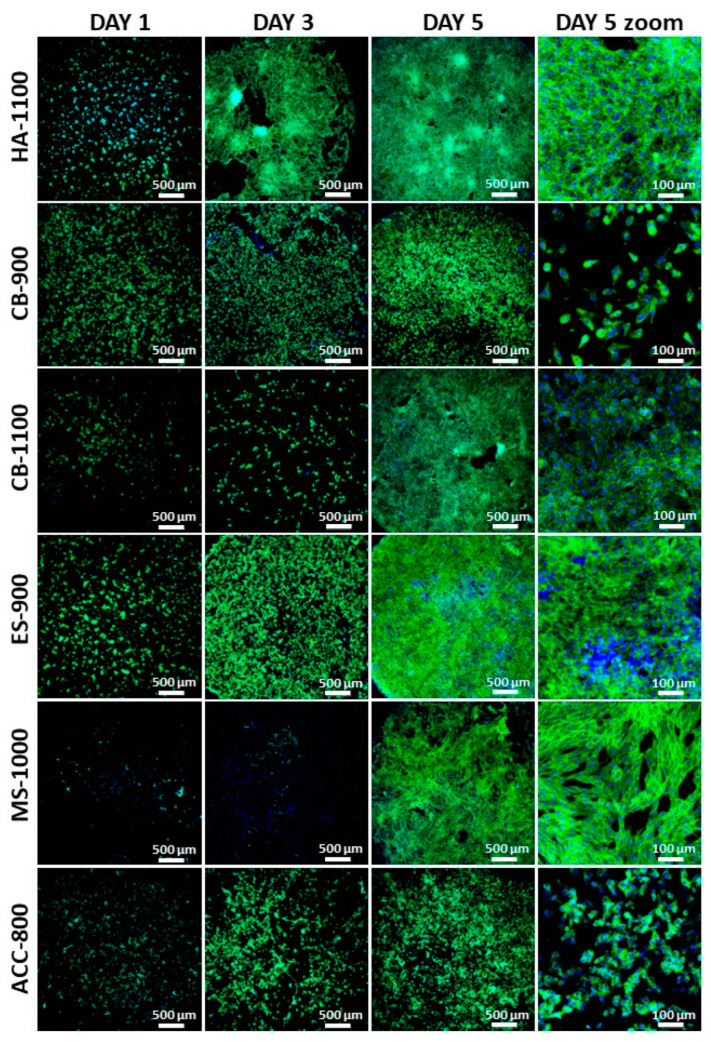
Confocal images of the cells adhered on the sintered pellets at day 1, 3 and 5 (the latter also with zoomed images).

Based on the cytotoxicity results, the same formulations were tested to study the preliminary cell adhesion at day 1, 3 and 5 after seeding, using the MG63 osteosarcoma cell line as cellular model. Cell adhesion was studied by confocal microscopy and the nuclei are stained in blue (DAPI staining) and cell cytoskeletons in green (I-Fluor488). As shown in Figure 5, at day 1, all conditions exhibited round shape cells and, among them, low cell adhesion can be detected in MS-1000 and CB-1100. At day 3, HA-1100 and ES-900 show a sensible improvement in the cell adhesion pattern, with an elongated cell morphology. Conversely, on CB-900, CB-1100, and ACC-800, at day 3, the adhesion is lower. Only the sample MS-1000 does not exhibit any differences at day 3 compared to day 1. Differently from what observed at previous times, at day 5, almost all the conditions show good cell adhesion, but with different shapes. In detail, evident cellular elongations are visible on HA-1100 sample at day 5 (clearer in the zoom, Figure 5). Cell–cell interconnections are easily visible and some cellular clusters can also be detected. Good cell adhesion and long-shaped cytoskeletons can be observed also on CB-1100, ES-900 and MS-1000, without the formation of clusters. However, as is clear from the zoom for MS-1000 at day 5 in Figure 5, the adhered cell’s morphology is clearly different from the other conditions since the cell cytoskeleton is more stretched and elongated. Lastly, CB-900 and ACC-800 are the only two samples exhibiting poor cell adhesion with the prevalence of round cellular shape.

In general, in all samples cell adhesion density increases upon culture as well as cell–cell interconnections, and cells are homogeneously distributed, with the exception of HA-1100, where clusters can be observed. If the samples obtained from natural resources are compared, ES-900 induces the fastest and highest cell density and CB-900 the lowest cell–cell interconnection. Considering the cell adhesion morphology, adhered cells are less spread on ACC-800 and CB-900, with the opposite effect being shown by CB-1100, ES-900, and MS-1100.

It is well known that ions play an important role in bone biology, cell spreading and adhesiveness. Among the different ions, besides zinc and calcium, magnesium can contribute to the adhesion and spreading of MG63 cells [77,78]. ICP analyses revealed the highest concentration of magnesium on ES-900 and this might explain the good cell adhesion pattern obtained on the eggshell-derived material. Conversely, ACC-800 showed the highest concentration of phosphorous and the presence of only sodium. The absence of magnesium among the other ions might have negatively affected cell adhesion and shape. In addition, although strontium is reported to have a beneficial effect on bone structural strength and osteoblast differentiation [78], in this preliminary test only early cell adhesion was studied. For this reason, even if cuttlebone-derived samples showed the highest strontium concentration, it cannot be directly correlated with the results obtained, and differentiation studies need to be performed. Moreover, the effect of HA and β-TCP phases on cell adhesion is difficult to correlate. In detail, in spite of the fact that low cell adhesion was detected in CB-900 composed of only the HA phase, the other samples, presenting the same HA phase, showed a good cell adhesion pattern, the opposite being revealed for β-TCP. Indeed, the best cell adhesion was observed on ES-900, which is a biphasic material (50/50 HA/β-TCP). Besides ionic and phase composition, the morphology of the surfaces where cells are seeded is probably relevant. The difference between the CB-900 and CB-1100 is visible via SEM imagery, and this might be sensed by cells in a different way, affecting their behavior.

## 4. Conclusions

Nanocrystalline hydroxyapatite derived from cuttlefish bones, eggshells, mussel shells and amorphous calcium carbonate can be synthesized and subsequently consolidated between 800 °C and 1100 °C to obtain bioactive calcium phosphate nanomaterials. The resulting crystalline phases (mainly HA, β-TCP and CPP) depend not only on the Ca/P molar ratio but also on the specific biogenic source, most probably due to the presence of different ionic species, like Mg^2+^ in eggshell-derived HA and Sr^2+^ in cuttlebone-derived HA.

The produced materials revealed the absence of any cytotoxic effect and good cell adhesion properties. Among the different calcium carbonate sources, eggshell-derived HA promotes the best cell adhesion and proliferation, which are comparable with those of pure HA, but without the formation of clusters. Cuttlebone- and mussel-derived HA also support cell adhesion well, while ACC- and cuttlebone-derived HA (sintered at 900 °C) show poor cell–cell interconnections. 

In conclusion, the results show that the considered biogenically and biomimetically derived materials are valid and compatible sources for producing materials suitable for bone regeneration application.

## Figures and Tables

**Table 1:** The parameters used during the mechanosynthesis process.

Raw Material	Label	Milling Time	Phosphate Provider	Drying Temperature
**DENSITY™**	ACC	30 min	(NH_4_)_2_HPO_4_	120 °C
**Eggshell**	ES	4 h	H_3_PO_4_	150 °C
**Mussel shell**	MS	4 h	(NH_4_)_2_HPO_4_	150 °C
**Cuttlebone**	CB	30 min	(NH_4_)_2_HPO_4_	120 °C

**Table 2:** The phase composition (wt%) of the raw materials, synthesized powders and sintered pellets determined with Rietveld analyses.

Raw Material		Synthesized Powder		Sintered Pellet	
Label	Phase Composition	Label	Phase Composition	Label	Phase Composition
ACC	100% amorphous CaCO_3_	ACC–HA	100% HA	ACC-800	~85% β-TCP, ~15% CPP
ES	100% calcite	ES–HA	100% HA	ES-900	~50% HA, ~50% β-TCP
MS	~70% calcite, ~30% aragonite	MS–HA	~93% HA, ~7% calcite	MS-1000	HA, <3% CaO
CB	100% aragonite	CB–HA	100% HA	CB-900	100% HA
				CB-1100	~90% β-TCP, ~5% HA, ~5% CaOH
sHA	-	-	-	sHA-1100	HA, <3% CaO

**Table 3:** The concentration of the fundamental elements and Ca/P molar ratio in the raw materials (CaCO_3_) and synthesized powders (HA) as determined by inductively coupled plasma/optical emission spectroscopy (ICP/OES) analysis.

	P	Ca/P Molar Ratio	Na		K		Mg		Sr	
	CaCO_3_	HA	CaCO_3_	HA	CaCO_3_	HA	CaCO_3_	HA	CaCO_3_	HA
**ACC**	2.0%	1.28 ± 0.02	1.5%	1.4%	<0.1%	-	-	-	<0.1%	<0.1%
**MS**	<0.2%	1.76 ± 0.02	0.3%	0.3%	<0.1%	-	0.1%	0.1%	0.1%	0.1%
**CB**	<0.2%	1.64 ± 0.02	0.7%	0.9%	0.1%	0.1%	<0.1%	0.1%	0.2%	0.2%
**ES**	<0.2%	1.58 ± 0.02	0.1%	0.1%	0.1%	0.1%	0.4%	0.3%	<0.1%	<0.1%
**sHA**	-	1.71 ± 0.02	-	<0.1%	-	<0.1%	-	<0.1%	-	<0.1%

**Table 4:** The bulk and relative density of the sintered pellets.

	ACC-800	MS-1000	CB-900	CB-1100	ES-900	sHA-1100
**Bulk density (g/cm^3^)**	2.19 ± 0.03	2.18 ± 0.08	2.33 ± 0.03	2.78 ± 0.03	2.06 ± 0.02	2.82 ± 0.08
**Relative density (%)**	71 ± 1	69 ± 2	74 ± 1	91 ± 1	66 ± 1	89 ± 2

## Data Availability

The data presented in this study are available on request from the corresponding author.
